# A Generalized Fisher Discriminant Analysis with Adaptive Entropic Regularization for Cross-Model Vibration State Monitoring in Wind Tunnels

**DOI:** 10.3390/s26020558

**Published:** 2026-01-14

**Authors:** Zhiyuan Li, Zhengjie Li, Xinghao Chen, Honghao Lin

**Affiliations:** China Aerodynamics Research and Development Center, Mianyang 621000, China

**Keywords:** vibration monitoring, Fisher Discriminant Analysis, health indicator, wind tunnel testing, aeroelasticity, entropic regularization, cross-model adaptation

## Abstract

The vibration monitoring of scaled models in wind tunnels is critical for aerodynamic testing and structural safety. The abrupt onset of flutter or other aeroelastic instabilities poses a significant risk, necessitating the development of real-time, model-agnostic monitoring systems. This paper proposes a novel, generalized health indicator (HI) based on an improved Fisher Discriminant Analysis (FDA) framework for vibration state classification. The core innovation lies in reformulating the FDA objective function to distinguish between stable and dangerous vibration states, rather than tracking degradation trends. To ensure cross-model applicability, a frequency-wise standardization technique is introduced, normalizing spectral amplitudes based on the statistics of a model’s stable state. Furthermore, a dual-mode entropic regularization term is incorporated into the optimization process. This term balances the dispersion of weights across frequency bands (promoting generalizability and avoiding overfitting to specific frequencies) with the concentration of weights on the most informative resonance frequencies (enhancing the sensitivity to dangerous states). The optimal frequency weights are obtained by solving a regularized generalized eigenvalue problem, and the resulting HI is the weighted sum of the standardized frequency amplitudes. The method is validated using simulated spectral data and flight data from a wind tunnel test, demonstrating a superior performance in the early detection of dangerous vibrations and the clear interpretability of critical frequency bands. Comparisons with traditional sparse measures and machine-learning methods highlight the proposed method’s advantages in trendability, robustness, and unique capability for cross-model adaptation.

## 1. Introduction

Vibration State Monitoring (VSM), also known as Condition Monitoring (CM), is a relatively new technology that has emerged over the past decade and has found numerous applications in various industries. For example, it is used for monitoring helicopters, aeroengines, and even rockets [1,2,3]. To address the issue of vertical tail wing oscillation that could lead to structural fatigue damage, a shortened service life, increased maintenance costs, and, ultimately, endanger flight safety, Liang et al. [4] proposed a multi-target point response prediction method for vertical tail wing flutter. This method can utilize wind tunnel data that do not fully meet the similarity criteria to predict the responses of full-scale models under multiple target flight conditions, thereby enhancing the utilization rate of the test data and prediction accuracy under multiple target flight conditions. To address the issue whereby the T-tail configuration is prone to special aerodynamic phenomena such as deep stall and flutter, and the issue whereby wind tunnel tests require high-precision dynamic scaling, Chen et al. [5] proposed a comprehensive optimization design method applicable to the dynamic proportional equivalent model of the T-tail structure with the aft fuselage. Jiao et al. [6] proposed a method for obtaining the aerodynamic loads of helicopter rotors by measuring the flapping moment using strain gauge sensors during flight. Compared with simulation calculations and wind tunnel tests, this method can provide more accurate load calculation results.

Long-term research and development, use, and maintenance experience have shown that these devices have the following characteristics: (1) the monitored objects all incur high design and maintenance costs; (2) they are heavily affected by either buffeting or flutter (commonly referred to as resonance in large CM articles); and (3) these devices require extensive wind tunnel testing before being put into use.

Although VSM has evolved rapidly, most systems still rely on human experience for early warning and post-maintenance. One is the potential for false alarms during flight, and the other is the lack of a method to assess their impact economically [7]. There is also an important reason for a conservative flight strategy, that is, the wind tunnel tests do not obtain the most comprehensive flight envelope possible; therefore, it is necessary to monitor the wind tunnel model tests. Wang et al. [8], based on advanced sensors and network technologies, monitored the components and parameters of the system in real time. Through the three-dimensional simulation method, they constructed a digital twin system for the wind tunnel system including the model, and combined data visualization methods with machine-learning technology to intelligently predict and diagnose faults and causes in the test data. Yang et al. [9] proposed a wind-tunnel-condition-monitoring surrogate model (POD-BPNN) integrating Proper Orthogonal Decomposition (POD) for data dimensionality reduction with Back Propagation Neural Networks (BPNNs). This methodology satisfies the precision and real-time requirements for structural/fluid field monitoring in wind tunnels. When deployed with an existing health management system, the online monitoring and predictive maintenance of the digital twin for the wind tunnel will be achievable.

However, the aforementioned methods either monitor the actual aircraft or the wind tunnel itself, but do not monitor the test models within the wind tunnel. Once the test model in the wind tunnel undergoes severe vibration and eventually disintegrates, it not only may cause irreparable damage to the wind tunnel, but also will delay the test progress and affect the upgrade of weapons and equipment. In addition, these devices require wind tunnel testing for sizing, layout design, flutter analysis, and before flight, but few methods provide the direct vibration monitoring of a single wind tunnel model, not to mention cross-model monitoring.

Wind tunnel testing is a cornerstone of aerospace engineering, enabling the evaluation of the aerodynamic performance and structural dynamics of scaled aircraft models [10]. A paramount concern during these tests is the occurrence of unforeseen, violent vibrations caused by phenomena such as flutter, buffeting, or flow separation. These events can lead to catastrophic model failure in seconds, jeopardizing safety and resulting in significant financial and time losses [11]. Consequently, the development of robust, real-time vibration-monitoring systems capable of providing early warnings is of the utmost importance. In addition, more accurate flight aerodynamic data can be obtained by wind tunnel tests without humans experiencing an emergency shutdown, which is of great significance to the flight of actual aircraft [12].

A significant challenge in this domain is the lack of a universal health indicator (HI) that can be applied across different aircraft models. The traditional vibration analysis often relies on the following:

(1) Time-domain metrics: Root Mean Square (RMS) and Kurtosis are two typical statistical HIs for VSM. For example, Malhi et al. [13] extracted the RMS and peak values from the wavelet coefficients as a signal preprocessing technique to predict the RUL of the bearings. However, the RMS offers monotonic trends; it is insensitive to incipient faults. Sparse metrics, such as kurtosis, are sensitive to impulsive transients but lack monotonicity and are highly susceptible to noise [14]. (2) Signal processing techniques: Methods like Spectral Kurtosis [15] and the Fast Kurtogram [16] are powerful for fault diagnosis in rotating machinery as they can identify informative frequency bands (IFBs). Wang et al. [17] consolidated these sparse methods into a generalized framework named the sum of the weighted normalized square envelope (SWNSE). However, their application for state classification in non-stationary, non-degrading wind tunnel environments is not straightforward. They often require expert interpretation and are not designed for cross-model generalization. (3) Machine-learning (ML) models: Machine-learning algorithms are widely used in the HI construction of PDA due to their strong nonlinear expression ability and data processing ability. Supervised and unsupervised learning algorithms [18,19] can fuse features for state classification. Shohanur et al. [20] present a practical, low-cost predictive maintenance system for subsonic wind tunnel facilities, leveraging edge-based sensor analytics and machine learning. The system integrates real-time data from vibration and thermal sensors deployed on key mechanical components, such as drive motors and axial fans, to detect early signs of wear, imbalance, and overheating. However, there are still some restrictions. First, the machine-learning-based VSM is irreversible; therefore, the trend of HI is expected to be monotonous. Based on the literature review, the monotonicity of HI is seldom considered mathematically. Second, existing machine-learning algorithms heavily rely on manual feature extraction based on some prior experience and expertise. Third, the HI of the machine-learning algorithm behaves like a black box, and its physical interpretation is difficult to achieve.

Although there are three methods, most of these methods are used for parts such as bearings or gearboxes, and a few methods are used to monitor the whole machine.

In general, the current approach lacks (1) a monitoring study of aircraft wind tunnel testing, (2) a proposal for a physically interpretable HI, and (3) a complete aircraft monitoring.

Fisher Discriminant Analysis (FDA) [21] is a classical method for dimensionality reduction and classification that maximizes the ratio of between-class scatter to within-class scatter. It has been widely used for feature selection and HI construction in prognostics and health management (PHM) [22,23]. However, its standard formulation is not directly suitable for the wind-tunnel-monitoring problem for two reasons: (1) its goal is typically multi-class classification or regression for degradation, not binary state alerting; and (2) it is not inherently designed for generalization across different units (models).

The main objective of this study is to develop a binary decision system for real-time discrimination between stable and dangerous states; and building a HI with good trend is the core means to achieve this goal.

Thus, this paper bridges this gap by proposing a novel, generalized FDA framework specifically designed for real-time, cross-model vibration state monitoring. The main contributions are the following: (1) Problem Reformulation: The FDA objective is reconfigured for binary state classification (Stable vs. Dangerous) instead of degradation assessment. (2) Cross-Model Adaptation: A model-specific standardization preprocessing step is introduced, allowing the same algorithmic core to be applied to different aircraft models. (3) Adaptive Entropic Regularization: A novel regularization term is added to the FDA objective function. This term leverages information entropy to balance the weight distribution, ensuring both sensitivity to critical resonances and robustness against model-specific spectral variations. (4) Interpretability and Diagnosis: The resulting optimal weight vector provides immediate physical insight into the most critical frequency bands responsible for the dangerous state, aiding in root cause analysis.

The rest of this paper is organized as follows: In Section 2, the theoretical basis of the FDA algorithm is briefly introduced, which theoretically demonstrates that the proposed method can be used to distinguish severe vibration from the normal operation of the wind tunnel model. In Section 3, the simulation data and three wind tunnel test data are used to verify the proposed method. Finally, the conclusions are provided in Section 4.

## 2. Theoretical Framework and Methodology

**Figure 1:**
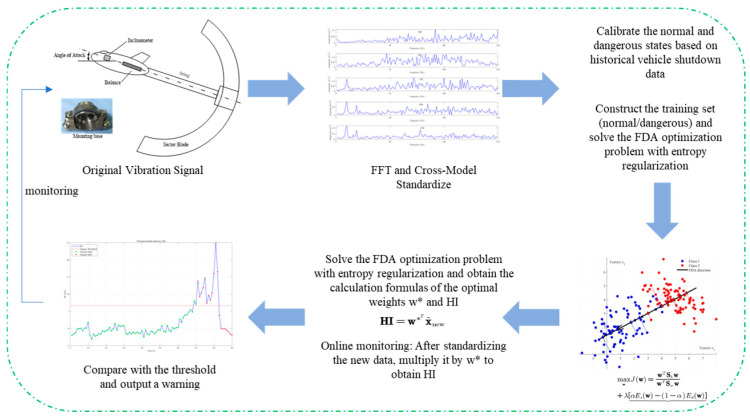
Overall workflow of the proposed method.

In this section, the basic idea of FDA is briefly introduced, and then the explicit expression of the optimal discriminant direction is given. Based on the evolution mechanism of wind tunnel test, a new proposition is proposed, which theoretically demonstrates that the optimal discriminant directions obtained by FDA, and the normal and abnormal spectrum amplitudes from normal operation to severe jitter data set can be used to locate information bands. The overall flowchart of the method proposed in this article is shown in Figure 1.

### 2.1. Problem Formulation and Preprocessing

Let X in $R^{d\times t_{i}}$ be a spectral matrix obtained from a wind tunnel test, where d is the number of frequency lines and t is the number of time observations. Each element $x_{ij}$ represents the amplitude at the ith frequency and the jth time epoch. Because, in wind tunnel testing, there are only normal operation and severe shaking stages (ignoring severe shaking will inevitably cause the model to fall apart and damage the wind tunnel, resulting in significant economic losses and delays in weapon equipment development), only binary classification problems are considered in this article. Thus, the time indices are partitioned into two classes:

$$\left|T_{\mathrm{normal}\;}=j\right|\;\mathrm{model}\;\mathrm{is}\;\mathrm{in}\;a\;\mathrm{stable}\;\mathrm{state}$$

$$|T_{\mathrm{danger}\;}=j|\;\mathrm{model}\;\mathrm{is}\;\mathrm{in}\;a\;\mathrm{dangerous}/\mathrm{vibratory}\;\mathrm{state}$$

To achieve cross-model adaptability, each frequency line is standardized based on the stable state data of the current model:

(1) $${\tilde{x}}_{ij}=\frac{x_{ij}-\mu_{i}}{\sigma_{i}+\epsilon }$$

where $\mu_{i}=\frac{1}{\left|T_{\mathrm{normal}\;}\right|}\sum_{j\in T_{\mathrm{nomal}\;}}{\tilde{x}}_{ij}$ and $\sigma_{i}=\sqrt{\frac{1}{\left|T_{\mathrm{normal}\;}\right|-1}\sum_{j\in T_{\mathrm{normal}\;}}{\left({\tilde{x}}_{ij}-\mu_{i}\right)}^{2}}$ are the mean and standard deviation of the ith frequency amplitude during the stable state, and ϵ is a small constant to avoid division by zero. This step ensures that the input data for all models has a similar statistical distribution, making the subsequent analysis model-agnostic. The standardized matrix is denoted by $\tilde{X}$.

### 2.2. Standard Fisher Discriminant Analysis

The goal of the FDA is to find a projection vector $w\in {\mathbb{R}}^{d}$ that maximizes the separation between the two classes. This is achieved by maximizing the Fisher criterion:

(2) $$S_{b}w=\lambda S_{w}w$$

The between-class scatter matrix $S_{b}$ captures the separation between class means:

(3) $$S_{b}=\left(\mu_{1}-\mu_{2}\right){\left(\mu_{1}-\mu_{2}\right)}^{T}$$

where $\mu_{1}=\frac{1}{|T\;\mathrm{normal}\;|}\sum_{j\in T_{\mathrm{normal}\;}}{\tilde{x}}_{j}$ is the mean vector of the stable class, and $\mu_{2}=\frac{1}{|T\;\mathrm{danger}\;|}\sum_{j\in T_{\mathrm{danger}}}{\tilde{x}}_{j}$ is the mean vector of the dangerous class. The within-class scatter matrix $S_{w}$ measures the internal variance of each class:

(4) $$S_{w}=\sum_{k=1}^{2}\sum_{j\in T_{k}}\left({\tilde{x}}_{j}-\mu_{k}\right){\left({\tilde{x}}_{j}-\mu_{k}\right)}^{T}$$

The optimal w is found by solving the generalized eigenvalue problem: $S_{b}w=\lambda S_{w}w$. The solution is the eigenvector corresponding to the largest eigenvalue.

### 2.3. Proposed Regularized Fisher Criterion

While the standard FDA provides good class separation, it lacks mechanisms for ensuring interpretable weight distributions and cross-model robustness. This paper proposes an enhanced objective function:

(5) $$\max_{w}J(w)=\frac{w^{T}S_{b}w}{w^{T}S_{w}w}+\lambda [(\alpha E_{s}(w)-(1-\alpha )E_{d}(w)]$$

where $\frac{w^{T}S_{b}w}{w^{T}S_{w}w}$ is the Fisher Discriminant Ratio and $\left[(\alpha E_{s}(w)-(1-\alpha )E_{d}(w)\right]$ is Dual-Mode Entropy Regularization, *λ* > 0 controls the overall regularization strength, α∈[0,1] balances the two entropy modes, $E_{s}(w)$ promotes weight dispersion (stable-state entropy), and $E_{d}(w)$ promotes the weight concentration on informative frequencies (danger-state entropy).

Stable-state Entropy (Es): To prevent overfitting to specific frequencies and enhance robustness, this paper encourages a dispersed weight distribution using the Shannon entropy:

(6) $$E_{s}(w)=-\sum_{i=1}^{d}p_{i}\ln\left(p_{i}\right)$$

where $p_{i}$ represents the normalized importance of the ith frequency:

(7) $$p_{i}=\frac{\left|w_{i}\right|}{\sum_{k=1}^{d}\left|w_{k}\right|}$$

The gradient of $E_{s}$ with respect to w is derived as follows. Let $S=\sum_{k=1}^{d}\left|w_{k}\right|$ be the sum of absolute weights. Then,

(8) $$\frac{\partial p_{i}}{\partial w_{j}}=\frac{\partial }{\partial w_{j}}\left(\frac{\left|w_{i}\right|}{S}\right)=\frac{\delta_{ij}\cdot \mathrm{sign}\left(w_{i}\right)\cdot S-\left|w_{i}\right|\cdot \mathrm{sign}\left(w_{j}\right)}{S^{2}}$$

where $\delta_{ij}$ is the Kronecker delta. This simplifies to the following:

(9) $$\frac{\partial p_{i}}{\partial w_{j}}=\frac{\mathrm{sign}\left(w_{i}\right)\cdot \delta_{ij}-p_{i}\cdot \mathrm{sign}\left(w_{j}\right)}{S}$$

Now, the gradient of $E_{s}$ becomes the following:

(10) $$\frac{\partial E_{s}}{\partial w_{j}}=-\sum_{i=1}^{d}\left(\frac{\partial p_{i}}{\partial w_{j}}\ln p_{i}+p_{i}\cdot \frac{1}{p_{i}}\cdot \frac{\partial p_{i}}{\partial w_{j}}\right)=-\sum_{i=1}^{d}\frac{\partial p_{i}}{\partial w_{j}}\left(1+\ln p_{i}\right)$$

Substituting the expression for $\frac{\partial p_{i}}{\partial w_{j}}$,

(11) $$\frac{\partial E_{s}}{\partial w_{j}}=-\frac{1}{S}\sum_{i=1}^{d}\left[\left(\mathrm{sign}\left(w_{i}\right)\delta_{ij}-p_{i}\mathrm{sign}\left(w_{j}\right)\right)\left(1+\ln p_{i}\right)\right]$$

This expands to the following:

(12) $$\frac{\partial E_{s}}{\partial w_{j}}=-\frac{1}{S}\left[\mathrm{sign}\left(w_{j}\right)\left(1+\ln p_{j}\right)-\mathrm{sign}\left(w_{j}\right)\sum_{i=1}^{d}p_{i}\left(1+\ln p_{i}\right)\right]$$

Recognizing that $\sum_{i=1}^{d}p_{i}=1$ and $\sum_{i=1}^{d}p_{i}\left(1+\ln p_{i}\right)=-E_{s}$, the final gradient can be obtained:

(13) $$\frac{\partial \pi }{\partial x_{i}}=-\frac{\sin\left(w_{i}\right)}{S}\left[\ln p_{i}+\cos\left(w_{i}\right)\right]$$

In vector form, it can be written as follows:

(14) $$\nabla E_{s}(w)=-\frac{1}{\sum_{k=1}^{d}\left|w_{k}\right|}\left[\mathrm{sign}(w){}^{\circ}\left(\mathrm{lnp}+E_{s}1\right)\right]$$

where ° denotes element-wise multiplication and 1 is a vector of ones.

Danger-state Entropy (Ed): To ensure a high sensitivity to the onset of vibration, we encourage the HI to be highly influenced by frequencies that change significantly in the dangerous state. This is promoted by minimizing the sum of absolute gradients:

(15) $$E_{d}(w)=\sum_{i=1}^{d}\left|{\left|\frac{\partial HI}{\partial w_{i}}\right|}_{T_{\;\mathrm{danger}\;}}\right|=\sum_{i=1}^{d}\frac{1}{\left|T_{\mathrm{danger}\;}\right|}\sum_{j\in T_{\mathrm{danger}}}{\tilde{x}}_{ij}$$

where $\sum_{i=1}^{d}\frac{1}{\left|T_{\mathrm{danger}\;}\right|}\sum_{j\in T_{\mathrm{danger}\;}}{\tilde{x}}_{ij}$ is the mean dangerous-state spectrum.

The gradient is straightforward:

(16) $$\nabla E_{d}(w)=\mathrm{sign}(d)$$

### 2.4. Complete Gradient of the Enhanced Objective Function

The total gradient of J(w) is as follows:

(17) $$\nabla J(w)=\nabla J_{\mathrm{fisher}\;}(w)+\lambda \left[\alpha \nabla E_{s}(w)-(1-\alpha )\nabla E_{d}(w)\right]$$

The Fisher discriminant ratio gradient requires careful derivation. Let

(18) $$J_{\mathrm{fisher}\;}=\frac{N}{D}=\frac{w^{T}S_{b}w}{w^{T}S_{w}w}$$

Then,

(19) $$\nabla J_{\mathrm{fisher}\;}=\frac{D\nabla N-N\nabla D}{D^{2}}$$

where $\nabla N=2S_{b}w$, and $\nabla D=2S_{w}w$.

Thus,

(20) $$\nabla J_{\mathrm{fisher}\;}=\frac{2}{D}\left(S_{b}w-J_{\mathrm{fisher}\;}S_{w}w\right)$$

### 2.5. Optimization Algorithm

This paper adopts the gradient ascent method with adaptive learning rate. The specific algorithm flow is detailed in Algorithm 1 below:
**Algorithm 1:** Enhanced FDA with Entropic Regularization(1) Input: $\tilde{X},T_{\mathrm{normal}},\;T_{\mathrm{danger}},\;\lambda ,\;\alpha ,\;\eta_{\max},\;\max\sim \mathrm{iter},\;\mathrm{tol}\;$
(2) Initialize:
Compute $S_{b}$, $S_{w}$Solve $S_{b}w=\lambda S_{w}w$ for initial $w^{\left(0\right)}$Normalize: $w^{\left(0\right)}\leftarrow \frac{w^{\left(0\right)}}{{\left|w^{\left(0\right)}\right|}_{2}}$
(3) For *k* = 0 to max~iter − 1:
Compute $J^{\left(k\right)}=J\left(w^{\left(k\right)}\right)$Compute $\nabla J^{\left(k\right)}=\nabla J\left(w^{\left(k\right)}\right)$Adaptive learning rate: $\eta =\min\left(\eta_{\max\;},\frac{0.1}{{\left|\nabla J^{\left(k\right)}\right|}_{2}}\right)$Update: $w^{\left(k+1\right)}=w^{\left(k\right)}+\eta \nabla J^{\left(k\right)}$Project to unit sphere: $w^{\left(k+1\right)}\leftarrow \frac{w^{\left(k+1\right)}}{{\left|w^{\left(k+1\right)}\right|}_{2}}$If $\left|J^{\left(k+1\right)}-J^{\left(k\right)}\right|<tol$: Break
(4) Output: $w^{*}=w^{\left(k+1\right)}$

### 2.6. Health Indicator

The Health Indicator for a new observation ${\tilde{X}}_{new}$ is as follows:

(21) $$HI=w^{*T}{\tilde{X}}_{new}$$

At this point, following the above steps, we find that HI can be displayed in real-time with only one training session, which is the core goal of this article: Cross-Model Vibration State Monitoring in Wind Tunnels. It should be noted that the cross model proposed in this article refers to a universal algorithm framework, rather than direct weight transfer, which can be achieved through standardized steps.

### 2.7. Theoretical Analysis

Based on the vibration patterns of the fixed strut of the aircraft from low Mach numbers to high Mach numbers, as well as the amplitudes of normal and abnormal frequency spectra, it is theoretically demonstrated that the optimal discriminant direction obtained from FDA can be used to locate the information frequency band.

**Theorem 1** 
(Convexity of Regularized Problem)**.** *The enhanced objective function* J(w) *is strictly concave in w under mild conditions.*

**Proof of Theorem 1.** 
The Fisher discriminant ratio $J_{\mathrm{fisher}\;}(w)$ is a generalized Rayleigh quotient known to be quasi-convex. The entropy term $E_{s}(w)$ is strictly concave in p, which is a linear transformation of $\left|w\right|$. The danger-state term $E_{d}(w)$ is linear in $\left|w\right|$. With proper choice of λ and α, the overall function becomes strictly concave, guaranteeing a unique global maximum. □

**Theorem 2** 
(Model Invariance)**.** *The standardized health indicator is invariant to affine transformations of input spectra.*

**Proof of Theorem 2.** 
Consider an affine transformation of the original spectra: $x_{ij}^{\prime }=a\cdot x_{ij}+b$. After standardization:

(22) $${\tilde{x}}_{ij}^{\prime }=\frac{a\cdot x_{ij}+b-\left(a\cdot \mu_{i}+b\right)}{a\cdot \sigma_{i}}=\frac{x_{ij}-\mu_{i}}{\sigma_{i}}={\tilde{x}}_{ij}$$

□

### 2.8. Computational Complexity

The algorithm’s complexity is dominated by the following:

- Eigenvalue decomposition: $O(d^{3})$ for the initial solution;
- Gradient computation: $O(d^{2})$ per iteration;
- Matrix operations: $O\left(d^{2}\cdot \left(\left|T_{\mathrm{normal}\;}\right|+\left|T_{\mathrm{danger}\;}\right|\right)\right)$.

For typical values (e.g., d=150, t=90), the algorithm converges in seconds on standard hardware, making it suitable for real-time applications.

This detailed mathematical derivation provides a rigorous foundation for the proposed method, ensuring both theoretical soundness and practical implementability.

## 3. Case Study

This section demonstrates how the proposed generalized Fisher discriminant analysis with adaptive entropic regularization works in two different scenarios. In addition, some famous approaches are provided for comparison to demonstrate how well the proposed methodology performs.

### 3.1. Simulation Test

**Figure 2:**
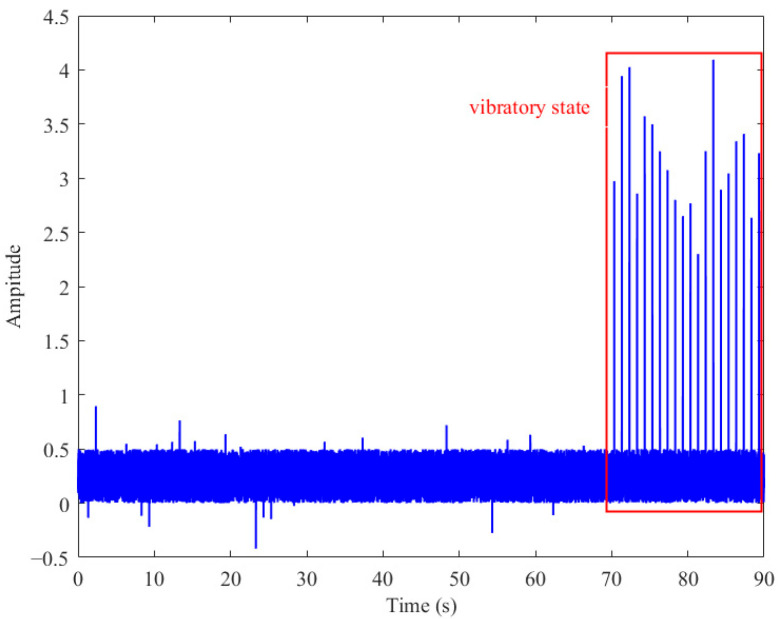
Analog spectrum failure signal.

To briefly verify the validity of the proposed method, a section of the spectrum data instead of the time data is given, where the sampling frequency of the spectrum data is 300 Hz and the sampling time is 90 s. To simulate the abnormal vibration of the model in the wind tunnel test, the last 20 s are set as the violent vibration stage, and the resonance frequency is set as 50 Hz. The spectrum simulation diagram is shown in Figure 2 below.

**Figure 3:**
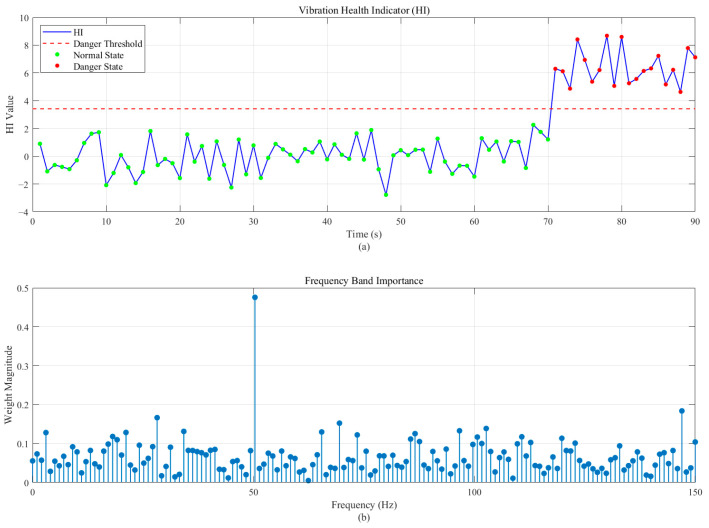
The proposed method applied to the performance of analog signal: (**a**) evolution of the Health Indicator (HI) over time; and (**b**) the optimized frequency weight vector $\left|w^{*}\right|$.

In order to verify the validity proposed by this method, the presented methodology with λ set to 0.2 and α set to 0.8 was applied to this analog signal. The proposed classification health indicator by using the optimized weights in Figure 3b is shown in Figure 3a, where a threshold can be calculated to confirm the exact time of the incipient fault by using a three-sigma rule. Based on the developed health indicator in Figure 3, it is demonstrated that file number 70 is the time of incipient fault initiation and an obvious degradation trend can be observed from the proposed health indicator to inform violent vibration. Thus, the application of the proposed method in CM and feature extraction has been verified separately. Further discussion of the parameter settings will be discussed in the following wind tunnel test data.

### 3.2. Description and Experimental Setup

**Figure 4:**
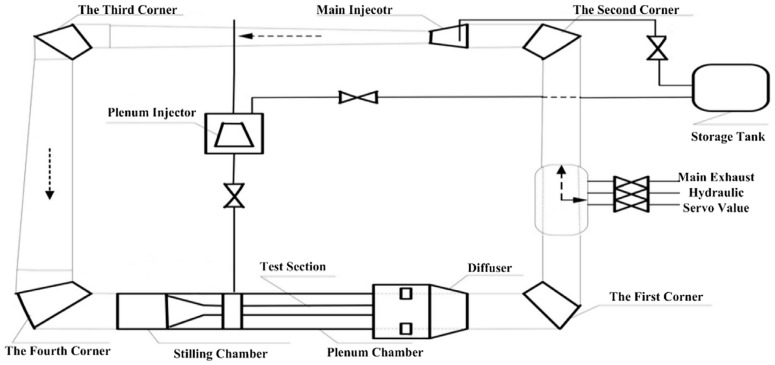
Diagram of transonic wind tunnel.

The 2.4 m × 2.4 m wind tunnel is a semi-circuit, intermittent, blow-down transonic wind tunnel capable of testing over a Mach number range from 0.3 to 1.2 [24]. A diagram of the transonic wind tunnel is showed in Figure 4. The direction of the arrow in the figure indicates the direction of airflow during the experiment. It has several test sections with different functions and large size [25], and possesses a dynamic data acquisition system that records signals with the synchronous scanning of all analog channels, so it is an ideal facility for vibration state monitoring.

**Figure 5:**
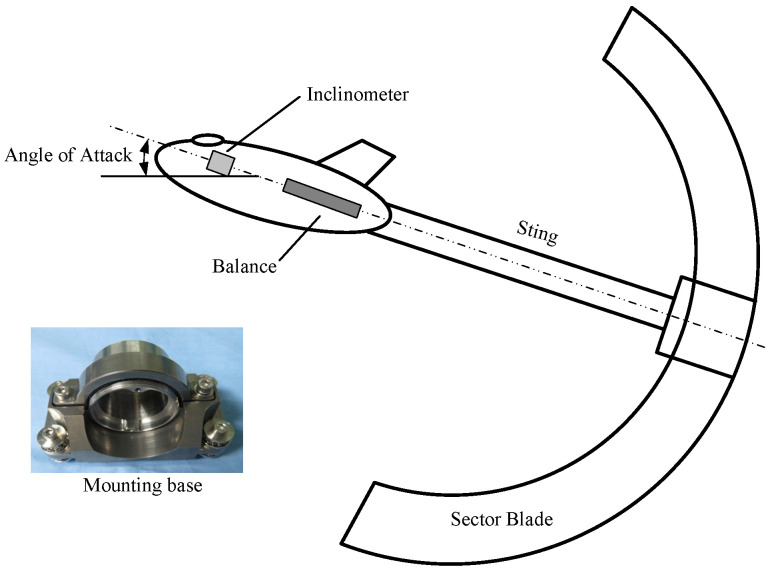
Schematic diagram of acceleration sensor installation and model support mechanism.

Time-domain data was collected from an accelerometer mounted inside a scaled aircraft model in a 2.4 m × 2.4 m wind tunnel. This data is taken from a piezometric test that requires simultaneous measurements of the model’s angle of attack, which is collected by an acceleration sensor built into the model. The built-in accelerometer in the model collects data. This accelerometer is a three-axis ICP accelerometer installed near the center of gravity of the model and its base and installation position inside the model are shown in Figure 5 below. To verify the effectiveness of the method proposed in this paper, three aircraft datasets are used in this method, including two emergency shutdown datasets and one normal operation dataset. The three aircrafts, respectively, use one emergency shutdown data as the training set, which is recorded as case 1, and the remaining two aircraft data as the test set, where the emergency shutdown data is case 2, and the normal operation data is case 3. In order to verify the effectiveness of the cross model mentioned in this article, case 2 and case 3 are the test train numbers for the same experiment at different Mach numbers and under different conditions (such as different wing states), respectively. The label of ‘dangerous’ state is based on a comprehensive judgment of wind tunnel operation logs, high-speed camera records, and model structural safety thresholds. Specifically, when the vibration acceleration of the model exceeds the pre-set engineering safety threshold and is accompanied by visible severe shaking, the data for that period is marked as ‘dangerous’.

**Figure 6:**
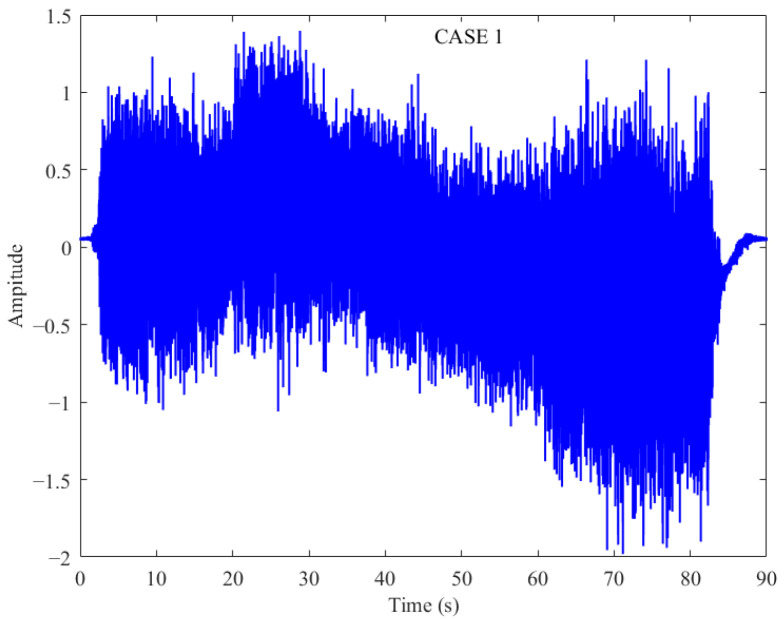
Time-domain diagram of case 1 model vibration.

The sampling frequency of the acceleration sensor of the three aircrafts is 300 Hz. The resulting spectrum had 150 frequency lines, and the case 1 data was recorded for 90 s. In the case 1 data, the model experienced severe vibrations leading to an emergency shutdown at the end of the test. The time-domain vibration wave characteristics are shown in Figure 6. Based on the operational logs and video evidence, the first 70 s were labeled as normal, and the final 20 s were labeled as dangerous.

First, to have a preliminary understanding of the aircraft over-vibration trend for vibration state monitoring, one time-domain metric and two sparse metrics are calculated to quantify this dataset by the following equations:

(23) $$X_{rms}=\sqrt{\frac{\sum_{i=1}^{N}X_{i}^{2}}{N}}=\sqrt{\frac{X_{1}^{2}+X_{2}^{2}+\cdots +X_{N}^{2}}{N}}$$

(24) $$\mathrm{Kurtosis}\;=\frac{\sum_{n=1}^{N}{\mathrm{SE}}^{2}[n]/N}{{\left(\sum_{n=1}^{N}{\mathrm{SE}}^{2}[n]/N\right)}^{2}}$$

(25) $$\mathrm{GI}=1-2\sum_{n=1}^{N}\frac{{\mathrm{SE}}_{\mathrm{order}\;}[n]}{\sum_{n=1}^{N}\mathrm{SE}[n]}\left(\frac{N-n+1/2}{N}\right)$$

where GI represents the Gini Index, SE[n] is the squared envelope of x[n], and ${\mathrm{SE}}_{\mathrm{order}}$ is the ordered SE[n] from the smallest to the largest.

**Figure 7:**
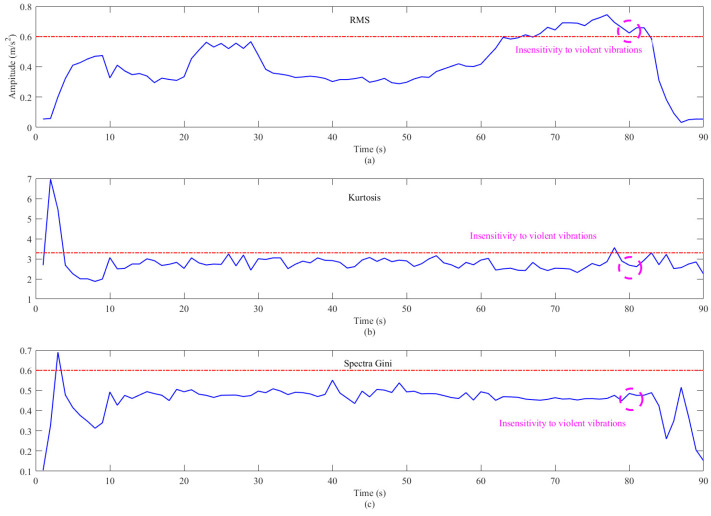
Three popular measures for vibration performance assessment: (**a**) RMS; (**b**) kurtosis; and (**c**) Gini Index.

The RMS is a typical time-domain indicator, while kurtosis and Gini Index are typical sparse indicators, and the quantification results by the three methods are shown in Figure 7. In this figure, the blue solid line represents the result calculated by the corresponding method every second, while the red dashed line represents the threshold value that should be obtained according to the corresponding method. These three indicators are not able to clearly detect the incipient violent vibration at file number 70; meanwhile, they also do not show a clear monotonic vibration trend. Under the premise of the failure of conventional methods, more advanced and general methods are required for wind tunnel model vibration monitoring.

**Figure 8:**
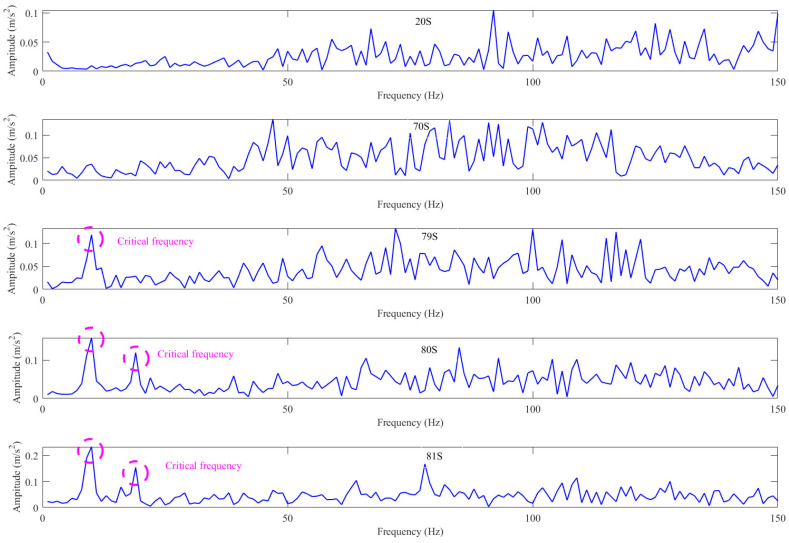
Fourier-transform spectrum of case 1 at different times 20, 70, 79, 80, and 81 s.

It can be observed that the spectrum data before (70 s before) the severe vibration and the normalized spectrum data after (20 s at last) the severe vibration (as shown in Figure 8 below) of the case 1 train demonstrates the following: in the normal operation stage, when the vibration state of the model is relatively stable, it is mainly affected by the high frequency; with the gradual vibration aggravation, the energy ratio of the low frequency to the whole frequency band starts to increase, and, with the time of the most severe vibration (79~80 s), the energy ratio reaches the maximum. It can also be seen from Figure 8 that the energy of the high frequency occupying the whole frequency band maintains a dynamic balance all the time. It can also be explained that the violent jitter of the model is mainly caused by a low frequency.

**Figure 9:**
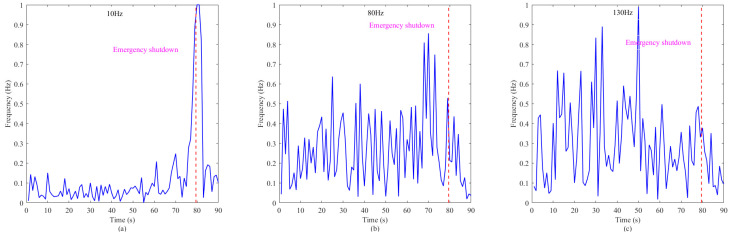
The temporal variation trends of three typical vibration frequencies in case 1: (**a**) 10 Hz; (**b**) 80 Hz; and (**c**) 130 Hz.

In addition, in this paper, low-frequency 10 Hz, high-frequency 80 Hz, and 130 Hz are separately selected for 90 s monitoring. The monitoring results are shown in Figure 9 below. The blue solid line represents the spectral value per second, while the red dashed line indicates the time points when dangerous vibrations occur. This also proves the aforementioned viewpoint.

### 3.3. Implementation and Results

The proposed algorithm was first implemented for case 1 with parameters λ=0.2, α=0.8, η=0.01, and κ=3. It should be noted that the weight of $\left|w^{*}\right|$ shown in case 1 is the absolute value, which is only measured as its contribution degree, and its vector of $\left|w^{*}\right|$ is actually positive and negative.

**Figure 10:**
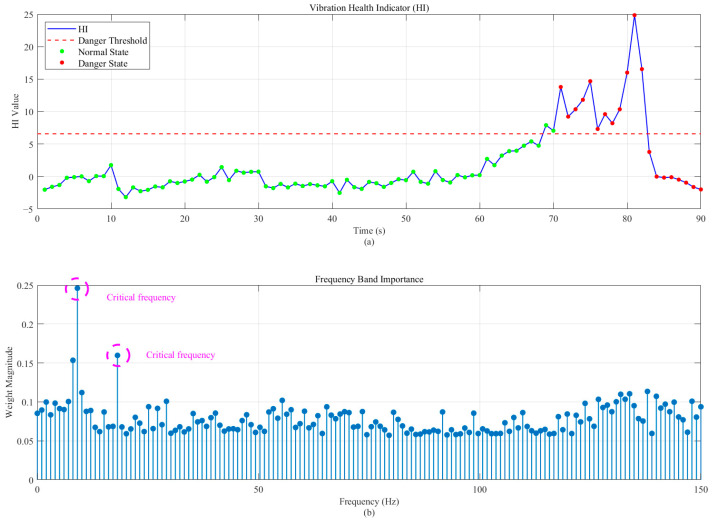
The proposed method applied to the performance of case 1: (**a**) evolution of the Health Indicator (HI) over time; and (**b**) the optimized frequency weight vector.

Figure 10a shows the resulting HI. The value remains stable and low during the normal operational period. It exhibits a sharp, monotonic increase upon the onset of the dangerous vibrational state, crossing the threshold and providing a clear alert signal. Figure 10b shows the optimized weights, which are sharply concentrated around 10 Hz. Compared with the three methods shown in Figure 7, it can be found that the method proposed in this paper has an excellent vibration characterization capability. This identifies the primary resonant frequency responsible for the instability, providing valuable diagnostic information to engineers.

**Figure 11:**
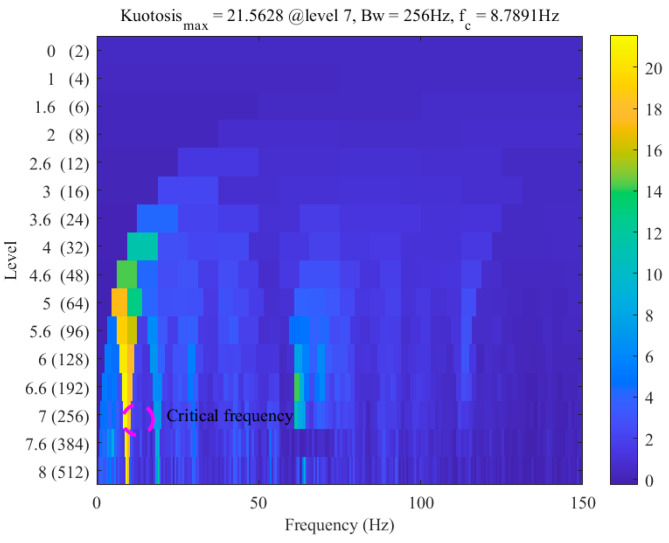
Incipient violent vibration diagnosis based on the fast Kurtogram.

To verify the validity of the frequency extracted by this method, Antoni’s Kurtogram was used, as shown in Figure 11 below. It can also be proven from the figure that the $\left|w^{*}\right|$ coefficient extracted by this method conforms to the analysis of the Kurtogram on the maximum frequency of vibration influence.

Thus far, the frequency contribution coefficient $\left|w^{*}\right|$ of the acceleration sensor is obtained from the training set, which applies to different models and different working conditions. For comparison, the data of case 2 and case 3 are also tested and verified with the same coefficient $\left|w^{*}\right|$.

**Figure 12:**
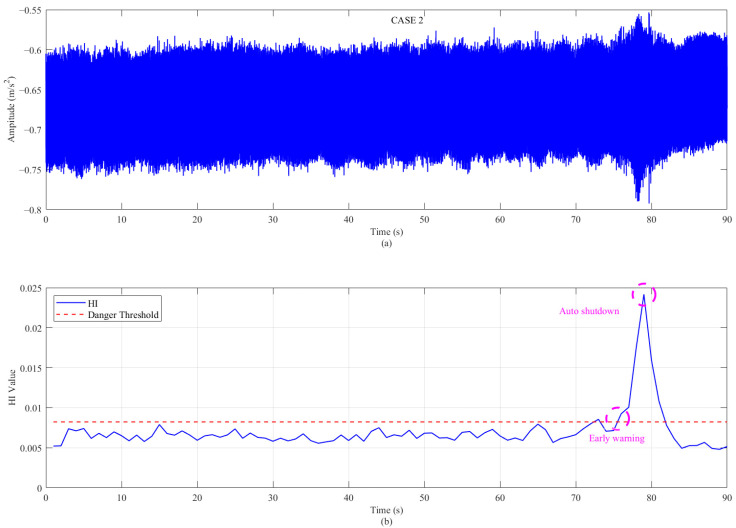
The proposed method applied to the performance of case 2: (**a**) evolution of the Health Indicator (HI) over time; and (**b**) the optimized frequency weight vector $\left|w^{*}\right|$.

**Figure 13:**
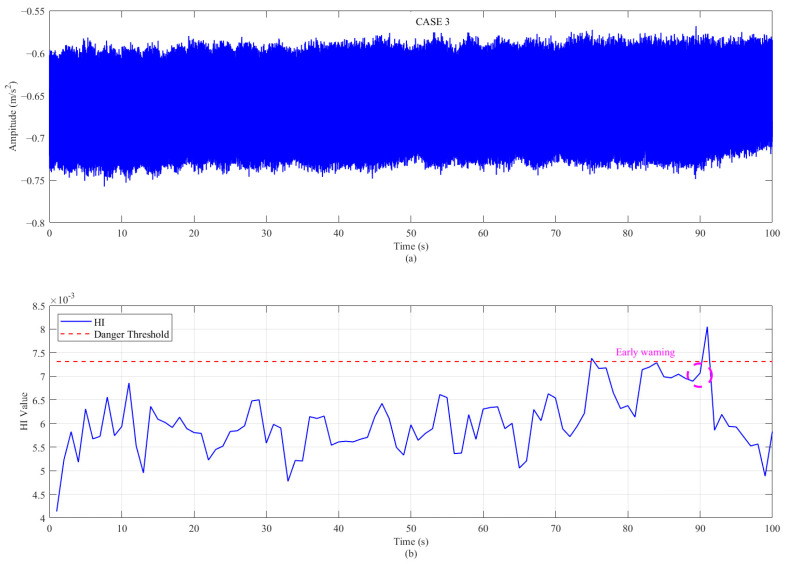
The proposed method applied to the performance of case 3: (**a**) evolution of the Health Indicator (HI) over time; and (**b**) the optimized frequency weight vector $\left|w^{*}\right|$.

Applying the same $\left|w^{*}\right|$ results in a trend chart as shown in Figure 12 and Figure 13 below. It can be clearly seen from the figure that the HI proposed in this paper has a good degradation trend in case 2, which is also an emergency shutdown. In contrast, the case 3 without an emergency shutdown only exceeds the threshold set by the manual at a certain time, and its mathematical distinction is minimal. In actual engineering application, case 3 can be used to give early warning to participants. The weight $\left|w^{*}\right|$ is learned from model A (case 1) and directly used for the HI calculation in operating conditions B (case 2) and C (case 3), proving the generality of the algorithm (standardization and framework) rather than the generality of the weight itself.

### 3.4. Quantitative Comparison with Other Methods

To demonstrate the superiority of the proposed method and quantitatively describe its ability in wind tunnel testing monitoring, the 1D Convolutional Neural Network (1D-CNN) and Support Vector Machine (SVM) methods will be compared. This article will compare the superiority of the methods using the signal-to-noise ratio, monotonicity, and detection delay. The detection delay is the time difference (in seconds) from the actual fault starting point to the first HI exceeding the threshold: the smaller, the better.

The formulae for the SNR and monotonicity are as follows:

(26) $$SNR=10*log10|(var(HI_{danger})/var(HI_{normal}))|$$

(27) Mon=|#(dHI/dt>0)−#(dHI/dt<0)|/(N−1)

Here, dHI/dt represents the derivative of HI with respect to time t (rate of change), #(dHI/dt>0) represents the number of positive derivatives (the increased part), #(dHI/dt<0) represents the number of negative derivatives (the decreased part), N represents the total number of data points, and (N−1) represents the number of derivatives (1 less than the number of data points).

It can be seen that, in the above formula, the energy of the dangerous state after the warning is used as a key component of the signal-to-noise ratio, which means that, in actual monitoring, it is hoped that this part can be as large as possible to make the warning signal distinguishable. In the monotonicity formula, only the trend from the warning time calculated by each algorithm to the actual shutdown time is calculated, and, the closer this part is to 1, the better. However, at the same time, the detection delay is expected to be negative because, during the actual operation of the wind tunnel, it is hoped that a warning can be issued 3–5 s in advance of the actual shutdown time.

These two methods also use the same case 1 standardized spectral data as input, and SVM (RBF kernel) and 1D-CNN as reference classifiers, and perform the same case 2 prediction. The comparison results are shown in Table 1 below.

In terms of the signal-to-noise ratio, the SNR of the method proposed in this paper is the highest (18.735), indicating that it has the strongest ability to extract dangerous state signals after early warning, and the highest discrimination between early warning signals and background noise, which is conducive to achieving more reliable anomaly identification in actual monitoring. The SNR of the SVM is the lowest (15.220), and it may be weak in noise suppression or feature extraction. The 1D-CNN lies between the two (16.890) and has a certain signal enhancement ability, but it is still inferior to the method proposed in this paper.

In monotonicity, the closer the monotonicity is to 1, the more stable and consistent the trend of the health index from the early warning to the actual shutdown is. The method proposed in this paper has the highest monotonicity (0.667), indicating that the trend after its early warning is closest to the ideal state, which is conducive to subsequent state assessment and decision-making. The SVM has the lowest monotonicity (0.512), and poor trend stability, and there may be fluctuations or misjudgments. The 1D-CNN is slightly superior to the SVM (0.598), but still has a certain gap compared with the method proposed in this paper.

A negative detection delay indicates that the warning occurs earlier than the actual fault, which meets the requirement of “warning 3 to 5 s in advance” in wind tunnel operation. The method proposed in this paper has the lowest delay (–4 s), fully meeting the requirements of early warning and leaving a safety margin. The SVM delay is –1.5 s. Although it is negative, the early warning lead is relatively small, and the response may not be timely enough in practice. The delay of the 1D-CNN is –2.3 s, which is superior to the SVM but still inferior to the method proposed in this paper.

### 3.5. Discussion

The FFT parameters used in this article (window function, window length, and overlap rate) are common choices for wind tunnel test data processing. Preliminary sensitivity analysis indicates that, within a reasonable spectral resolution range (such as frequency resolution between 0.5–2 Hz), the core resonance frequency and HI trend obtained by the proposed method remain stable. Extreme parameter settings may result in the loss of detailed information or noise amplification.

The proposed method successfully identified the dangerous state with a high signal-to-noise ratio in the HI trajectory. The entropic regularization effectively balanced the weight distribution, avoiding the noise sensitivity common to kurtosis-based measures. The clear identification of the resonance band demonstrates the method’s interpretability, a significant advantage over “black-box” ML approaches. The initial standardization step is the key to cross-model adaptation, as it removes model-specific amplitude characteristics, allowing the core algorithm to focus on the relative changes in spectral energy.

**Figure 14:**
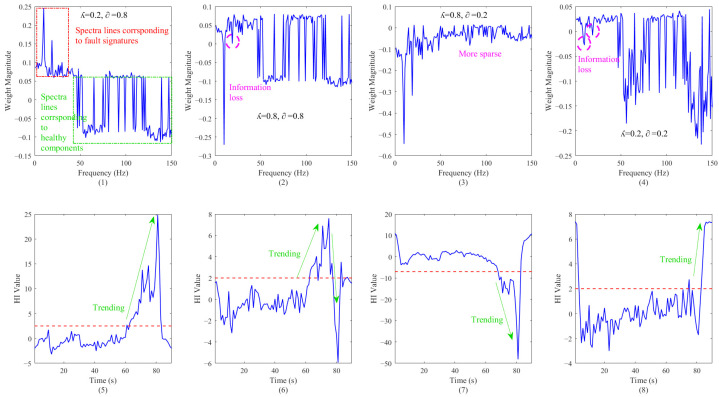
The impact of different α and λ values on monitoring: (**1**–**4**) the optimized frequency weight vector $\left|w^{*}\right|$ under different α and λ values; and (**5**–**8**) evolution of Health Index (HI) over time under different α and lambda λ.

As previously mentioned, $E_{s}(w)$ and $E_{d}(w)$ use different approaches to constrain the weight distribution, making it both generalized and sensitive. To explore the impact of different λ and α values on monitoring, this paper sets the parameters as shown in Figure 14 below for comparison against the data of the case 1 train number.

The $\left|w^{*}\right|$ values shown in Figure 11 are all original $\left|w^{*}\right|$ values, not their absolute values. According to the viewpoint mentioned in paper [26], negative $\left|w^{*}\right|$ weights represent healthy components (in this paper, the frequency that causes severe vibrations), while positive $\left|w^{*}\right|$ weights represent the corresponding fault signatures (in this paper, the frequency that causes severe vibrations). As shown in (1) and (5) of the above figure, when λ is set to 0.2 and α 0.8, this health indicator can effectively warn of severe vibrations in the model. In this figure, all red dashed lines represent the monitoring results obtained under different alpha and lambda settings, which are the thresholds set for these settings.

Figure 2 and Figure 6 increase the weight of $E_{s}(w)$ and $E_{d}(w)$ in the entire $\left|w^{*}\right|$ by increasing the λ. It can be observed that there is no significant change in the direction of the spectrum lines corresponding to the health components, but it leads to the reversal of the direction of the spectrum lines corresponding to the fault signals and the loss of some key information at low frequencies. When λ is set to 0.8 and α is also set to 0.8, the trend of this health indicator is poor and cannot be used for model monitoring in actual wind tunnel tests.

Similarly, in Figure 4 and Figure 8, when λ is set to 0.2 and α is also set to 0.2, due to the properties of $E_{d}(w)$, all weights begin to concentrate towards the frequency of normal operation, resulting in the entire $\left|w^{*}\right|$ becoming less sparse. And, at this time, the health indicator’s indication of key frequencies is not strong.

In Figure 3 and Figure 7, when λ is set to 0.8 and α is set to 0.2, the proportion of this entropy index to the entire index increases. Due to the properties of $E_{s}(w)$, all weights begin to concentrate towards the dangerous frequency, resulting in the entire $\left|w^{*}\right|$ becoming sparse. At this point, the indicator can still effectively indicate the most critical frequency that affects vibration, and also has a good tendency towards severe vibration.

*λ* > 0 controls the overall regularization strength, α∈[0,1] balances the two entropy modes, $E_{s}(w)$ promotes weight dispersion (stable-state entropy), and $E_{d}(w)$ promotes the weight concentration on informative frequencies (danger-state entropy).

According to the results of different settings of λ and α, they should have a negative correlation in use; otherwise, it will lead to missing information or a lack of prominent features in the key frequency band. Even for a set of parameters that are negatively correlated, in practical use, this article still recommends selecting health indicators with upward trends to facilitate monitoring by all personnel.

## 4. Conclusions

This paper presented a novel generalized FDA framework for real-time vibration state monitoring in wind tunnels. The method fundamentally reorients FDA from degradation tracking to binary state classification. Its two primary innovations—model-specific spectral standardization and dual-mode entropic regularization—directly address the critical challenges of cross-model applicability and robust feature weighting.

The case study demonstrated that the algorithm provides a clear, monotonic health indicator that reliably signals the onset of dangerous vibrations while also pinpointing the responsible resonant frequency bands. This combination of high detectability and physical interpretability makes it a powerful tool for wind tunnel engineers.

This method requires a small amount of labeled normal state data and known dangerous state data during the training phase. In actual wind tunnel testing, normal state data is easy to obtain, while hazardous state data may come from historical test records or high-risk operating conditions predicted through finite element analysis/Fluid Structure Interaction simulation. Future work will explore initialization strategies based on unsupervised or semi-supervised learning and focus on automating the selection of parameters λ and α, and validating the method on a larger dataset involving multiple, distinct aircraft models.

## Figures and Tables

**Table 1:** Performance comparison table of different methods.

Methods	SNR	Monotonicity	Detection Delay
This paper	18.735	0.667	−4
SVM	15.220	0.512	−1.5
1D-CNN	16.890	0.589	−2.3

## Data Availability

The original contributions presented in the study are included in the article; further inquiries can be directed to the corresponding author.

## Abbreviations

The following abbreviations are used in this manuscript:

VSM	Vibration State Monitoring
CM	Condition Monitoring
HI	Health Indicator
PHM	Prognostics and Health Management
FDA	Fisher Discriminant Analysis
RMS	Root Mean Square
GI	Gini Index
SNR	Signal-to-noise Ratio
1D-CNN	1D Convolutional Neural Network
SVM	Support Vector Machine
